# Qualitative evaluation of spatial complementarity between renewable energy resources with complementarity roses

**DOI:** 10.1016/j.mex.2019.04.005

**Published:** 2019-04-09

**Authors:** Alfonso Risso, Alexandre Beluco, Rita de Cássia M. Alves

**Affiliations:** a Centro de Pesquisas em Sensoriamento Remoto e Meteorologia (CEPSRM), Universidade Federal do Rio Grande do Sul (UFRGS), Porto Alegre, Rio Grande do Sul, Brazil; b Instituto de Pesquisas Hidráulicas (IPH), Universidade Federal do Rio Grande do Sul (UFRGS), Porto Alegre, Rio Grande do Sul, Brazil

**Keywords:** Method for qualitative evaluation of spatial complementarity between renewable energy resources with complementarity roses, Renewable energy, Energetic complementarity, Hybrid energy systems, Spatial complementarity, Complementarity roses, Maps of complementarity

## Abstract

Energetic complementarity is a subject that has been concentrating more and more attention of the researchers around the world in the last years, a concept that can be applied both in energy planning and in operation of energy systems based on renewable energy resources. Spatial complementarity is the energetic complementarity evaluated between two renewable resources in different locations and, as well as the complementarity evaluated between resources in the same location, has three components: time-complementarity, energy-complementarity and amplitude-complementarity. At the same site, however, complementarity assessment can involve multiple resources simultaneously, and the study of these circumstances requires appropriate tools to handle such information.This method paper describes a method to build complementarity roses expressing the spatial complementarity between two or more renewable energy resources throughout a region, appropriate for the expression of this complementarity through maps.

## Graphical abstract

**Figure:**
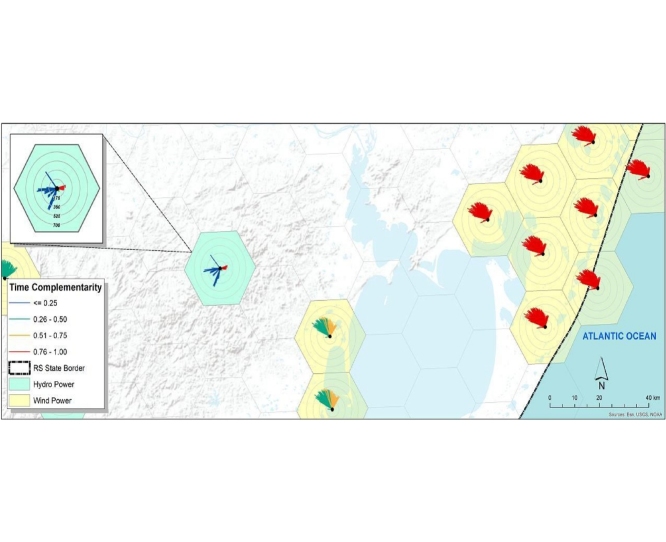

• The method allows the graphic characterization of the spatial complementarity along a region with the use of maps.
• The complementarity rose, inspired by the compass-rose, expresses a reasonable amount of information in one graphic symbol.
• A complementarity rose can be built with components or with total complementarity, determined with different methods.

**Specifications Table**

**Table:** 

Subject Area:	*Energy*
More specific subject area:	*Renewable Energy – Hybrid Energy Systems*
Method name:	*Method for qualitative evaluation of spatial complementarity between renewable energy resources with complementarity roses*
Name and reference of original method:	*Complementarity roses evaluating spatial complementarity in time between energy resources. Risso et al.* [2]*. Energies (2018), v.11, n.7, #1918.*
Resource availability:	*N/A*

## Method details

### Background

The subject of the complementarity between renewable energy resources has been attracting the attention of a growing number of researchers around the world in recent years. The work of Beluco et al. [1] suggested a dimensionless index for the evaluation of the complementarity between two or more renewable resources in the same place. One of the challenges is the evaluation of the complementarity between renewable resources in different places and the work of Risso et al. [2] proposes a qualitative evaluation of this type of complementarity, denominated as spatial, with the use of complementary roses. Ref. [3] presents a more extensive discussion on the subject, also advancing on issues related to the performance of hybrid systems based on complementary resources. Ref. [4] relates the reliability of a hybrid system with the complementarity between energy resources and Refs. [[5], [6], [7], [8]] present practical situations related to complementarity. This method article summarizes a method for the qualitative evaluation of spatial complementarity by means of complementarity roses.

### Method

The method for qualitative evaluation of spatial complementarity between renewable energy resources with complementarity roses consists of the following steps:

1 Establish the extension of the study to be performed with the application of complementary roses to evaluate spatial complementarity, specifically determining the region to be covered by the study and the type of complementarity that will be determined (total complementarity or some of its components, complementarity between energy supplies provided by power plants or complementarity between energy potentials).
2 Select the power plants or energy potentials that will be included in the determination of complementarity within the region already delimited for the study. Obtain data series of power supplied (for plants in operation) or energy availability (for sites with energetic potential) that will be considered in the determination of complementarity. Select coincident time periods in these data series and build monthly average data series.

Note. This step suggests the use of monthly data, for the practicality of working with smaller amounts of data, since the evaluation of complementarity itself will require a great amount of information. Clearly, the analysis can be performed with daily data and with hourly data and even with studies differentiating results by seasons, if convenient. Probably the more detailed resolutions will be more convenient for more detailed studies, closer to the definition of energy resource management plans.

3 Establish a network of hexagonal cells over the region chosen for the study, identifying cells containing power plants in operation or sites with energetic potential and recording the distances between these cells. For cells with more than one plant or one energy potential, the data series must be summed resulting in one data series per cell per resource.The distances between cells must be measured between the center points of each hexagonal cell.

Note. The size of the hexagonal cells must be established from the full extent of the network used for the analysis and the individual size of the cells so that the complementary roses have dimensions that allow their correct visibility and a comprehensive analysis of spatial complementarity. Ref. [2] shows an example of a hexagonal network determination.

4 Determine the complementarity (as established in step # 1) for each cell containing some existing or potential plant, comparing it with all other cells containing plants, recording in a complementarity rose for this cell the different values of complementarity obtained as a function of the direction and the distance between the compared cells.Complementary roses must be determined according to the next two steps, as the cell under analysis contains only one or more than one renewable resource.

Note. Complementarity can be determined in different ways, including with the method described in Ref. [9]. This reference describes a simplified method for determining the total complementarity and its components from monthly average data series.

5 If the cell under analysis contains only one renewable resource, the type of this resource must be indicated with the color assigned to that cell. From their central point, lines should be drawn in the respective directions to the other cells containing plants, with the respective colors defined by the complementarity intensities and with the respective lengths indicating the distances to those cells.The center of the cell should be marked with a single dot.

**Fig. 1:**
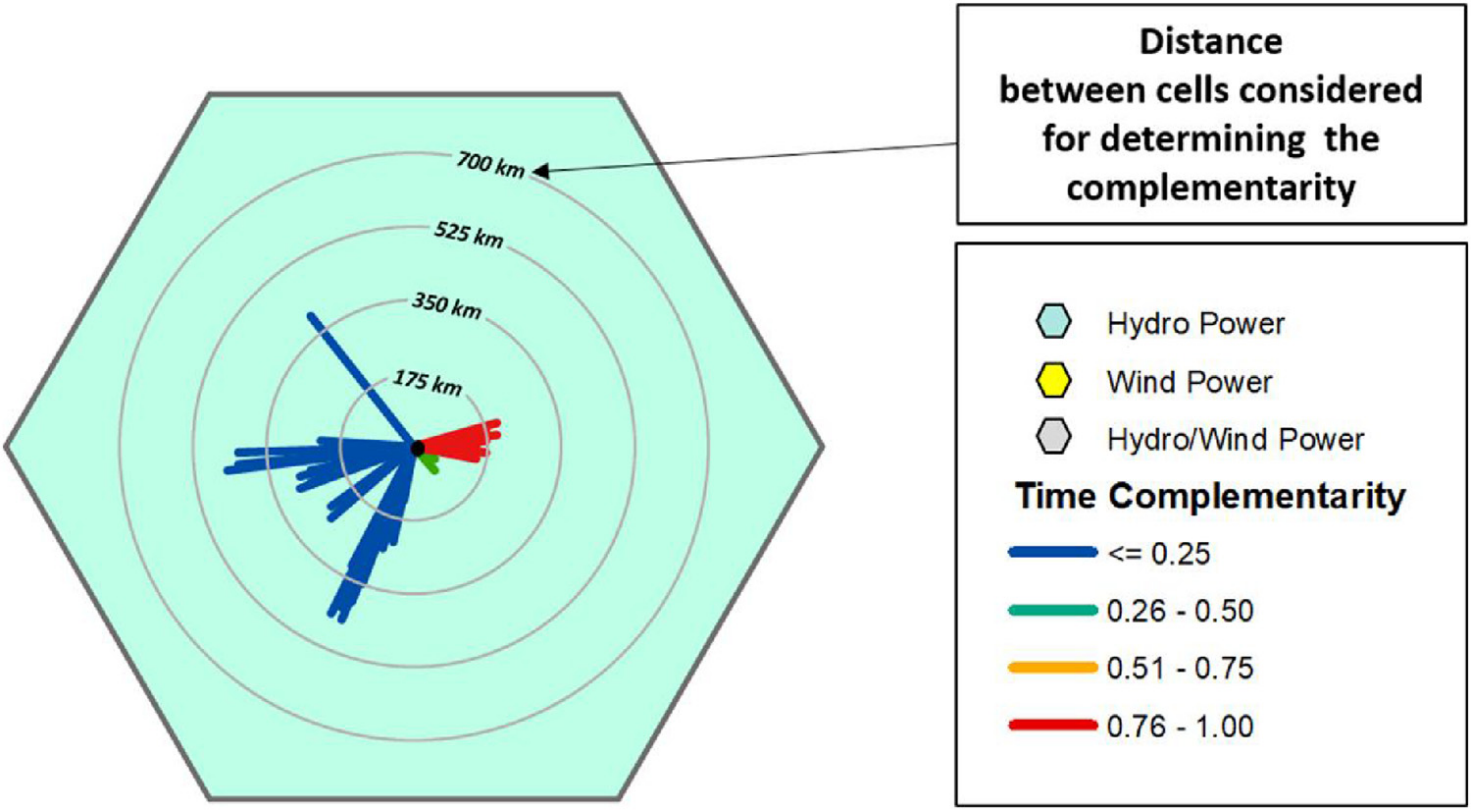
Model for determining complementarity rose for a hexagonal cell with only one renewable energy resource.

**Fig. 2:**
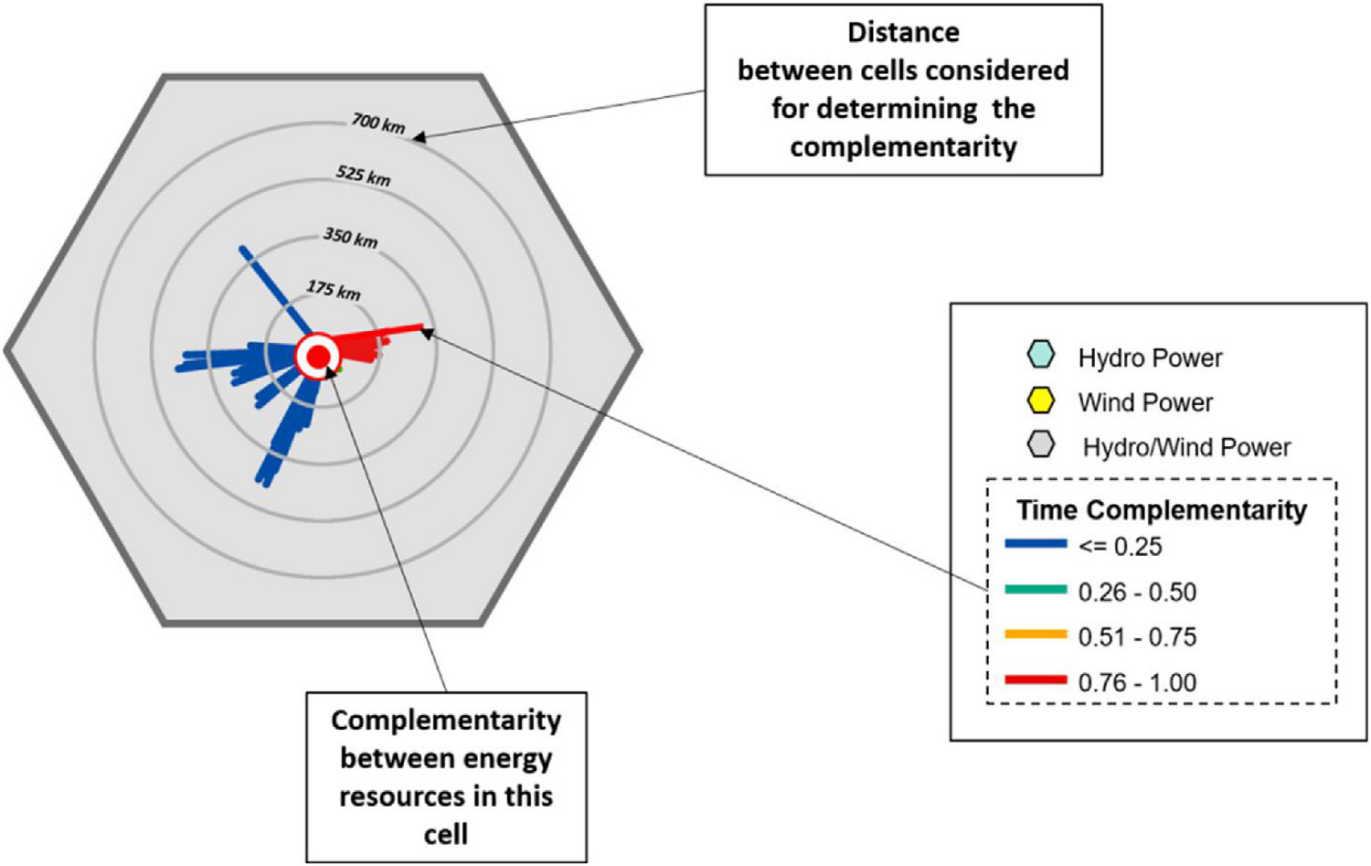
Model for determining complementarity rose for a hexagonal cell with more than one renewable energy resource.

Note. Fig. 1 shows an example of a cell containing only one energy resource. The caption indicates the color of the feature present in that cell. The circles indicate the distances and the colors of the lines indicate (according to the corresponding legend) the intensities of complementarity in each direction. Fig. 1, Fig. 2 together can clarify the construction of these cells.

6 If the cell under analysis contains more than one renewable resource, the cell should be filled with gray and the center point should be thicker than a single point and its color should indicate the intensity of temporal complementarity between the energy resources present in this cell. As in the previous step, from their central point, the lines should be drawn in the respective directions to the other cells containing plants, with the respective colors defined by the complementarity intensities and with the respective lengths indicating the distances for those cells.

(Figure reproduced from Ref. [2]).

(Figure reproduced from Ref. [2]).

Note. Fig. 2 shows an example of a cell containing more than one energy resource. The central point indicates the intensity of complementarity between the energy resources present in this cell. The circles indicate the distances and the colors of the lines indicate (according to the corresponding legend) the intensities of complementarity in each direction. Fig. 1, Fig. 2 together can clarify the construction of these cells.

7 Superpose the hexagonal network with the cells indicating their complementary roses on the map of the region under analysis to give a graphic expression of spatial complementarity.Evaluate if the final result allows a good expression of the complementarity, eventually eliminating some cells from the analysis to compose an easy-to-read map.

**Fig. 3:**
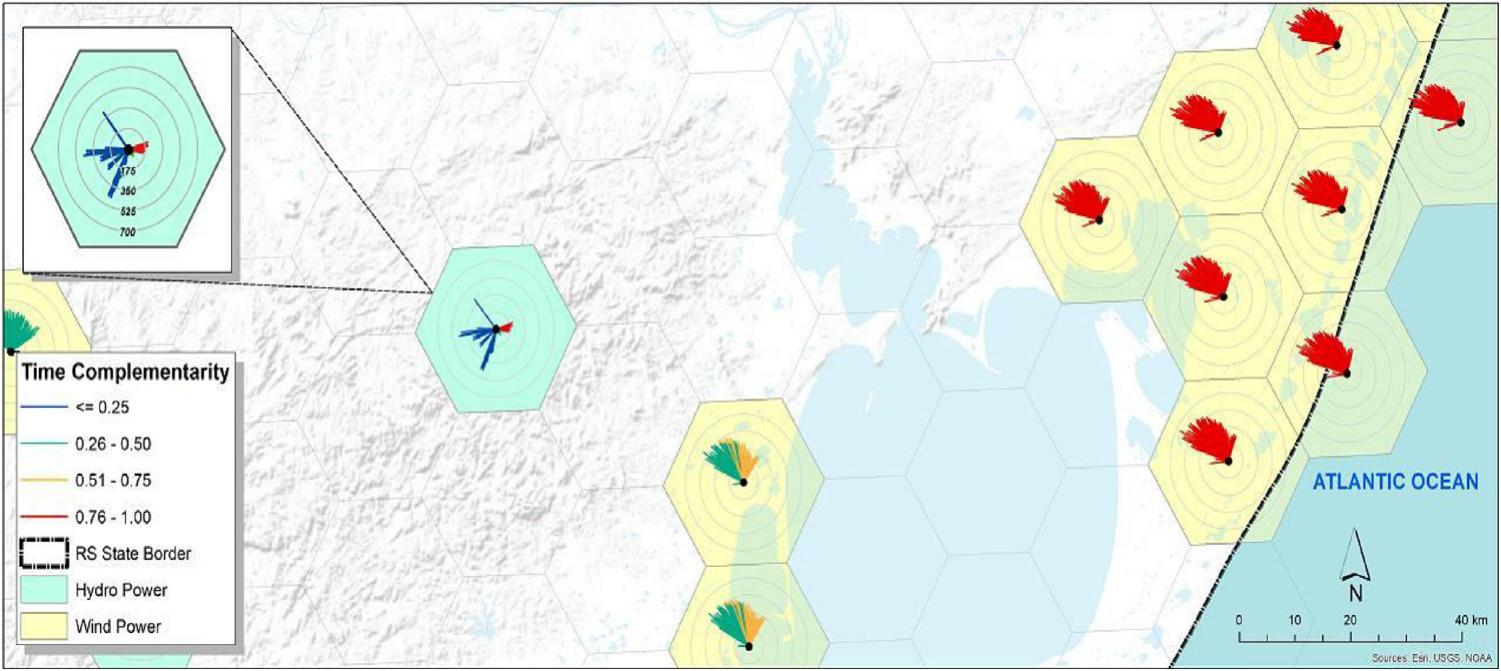
Map of spatial complementarity.

Note. Fig. 3 shows an example of a spatial complementarity map in which a hexagonal network with complementary roses built in the cells containing renewable energy resources was superimposed on the map of the region under analysis.
